# Supplementary material for: The Stability of Hop (Humulus lupulus L.) Resins during Long-Period Storage
Source: Plants (Basel). 2023 Feb 18;12(4):936. doi: 10.3390/plants12040936 (PMC9960943; doi:10.3390/plants12040936)
Supplement: Supplementary file 1 [file plants-12-00936-s001.zip › SUPPLEMENTARY TABLES.pdf]

Table S1: The content of co-humulone. (n+ad)-humulone and % of co-humulone in alpha-acids in hop cones.

	time	co-humulone				(n+ad)-humulone				% co-humulone in alpha-acids			
Variety/Conditions		A	B	C	D	A	B	C	D	A	B	C	D
Celeia cones	0	0.99 ± 0.02	0.99 ± 0.02	0.99 ± 0.02	0.99 ± 0.02	2.50 ± 0.04	2.50 ± 0.04	2.50 ± 0.04	2.50 ± 0.04	28.3 ± 0.9	28.3 ± 0.9	28.3 ± 0.9	28.3 ± 0.9
	6	0.83 ± 0.01	0.80 ± 0.01	0.74 ± 0.01	0.55 ± 0.01	1.99 ± 0.04	1.94 ± 0.03	1.87 ± 0.03	1.4 ± 0.03	29.4 ± 0.8	29.3 ± 0.8	28.4 ± 0.8	28.2 ± 0.7
	12	0.79 ± 0.01	0.67 ± 0.01	0.58 ± 0.01	0.3 ± 0.01	1.91 ± 0.03	1.64 ± 0.03	1.47 ± 0.03	0.73 ± 0.01	29.3 ± 0.8	28.9 ± 0.8	28.1 ± 0.7	29.3 ± 0.7
	18	0.77 ± 0.01	0.63 ± 0.01	0.52 ± 0.01	0.23 ± 0.01	1.88 ± 0.03	1.54 ± 0.03	1.25 ± 0.02	0.55 ± 0.01	29.1 ± 0.8	28.9 ± 0.8	29.4 ± 0.7	30.0 ± 0.6
	24	0.67 ± 0.01	0.50 ± 0.01	0.37 ± 0.01	0.13 ± 0.01	1.65 ± 0.03	1.19 ± 0.02	0.95 ± 0.02	0.30 ± 0.01	28.9 ± 0.8	29.4 ± 0.7	28.3 ± 0.7	30.8 ± 0.6
Auora cones	0	2.70 ± 0.05	2.70 ± 0.05	2.70 ± 0.05	2.70 ± 0.05	9.48 ± 0.17	9.48 ± 0.17	9.48 ± 0.17	9.48 ± 0.17	22.2 ± 1.2	22.2 ± 1.2	22.2 ± 1.2	22.2 ± 1.2
	6	2.54 ± 0.04	2.43 ± 0.04	2.29 ± 0.04	2.16 ± 0.04	8.98 ± 0.16	8.75 ± 0.16	8.12 ± 0.15	7.50 ± 0.13	22.1 ± 1.2	22.0 ± 1.1	22.4 ± 1.1	22.4 ± 1.1
	12	2.43 ± 0.04	2.20 ± 0.04	2.13 ± 0.04	1.39 ± 0.02	8.55 ± 0.15	7.72 ± 0.14	7.44 ± 0.13	4.83 ± 0.09	22.1 ± 1.1	22.3 ± 1.1	22.3 ± 1.1	22.6 ± 0.9
	18	2.39 ± 0.04	2.11 ± 0.04	1.95 ± 0.03	1.03 ± 0.02	8.41 ± 0.15	7.47 ± 0.13	6.66 ± 0.12	3.43 ± 0.06	22.1 ± 1.1	22.6 ± 1.1	23.1 ± 1.1	22.7 ± 0.8
	24	2.26 ± 0.04	1.79 ± 0.03	1.75 ± 0.03	0.70 ± 0.01	7.88 ± 0.14	6.38 ± 0.11	6.25 ± 0.11	2.35 ± 0.04	22.3 ± 1.1	21.9 ± 1.0	23.0 ± 1.0	22.9 ± 0.7
Bobek cones	0	0.93 ± 0.02	0.93 ± 0.02	0.93 ± 0.02	0.93 ± 0.02	2.55 ± 0.05	2.55 ± 0.05	2.55 ± 0.05	2.55 ± 0.05	26.7 ± 0.8	26.7 ± 0.8	26.7 ± 0.8	26.7 ± 0.8
	6	0.89 ± 0.02	0.76 ± 0.01	0.76 ± 0.01	0.75 ± 0.01	2.38 ± 0.04	2.06 ± 0.04	1.93 ± 0.03	1.94 ± 0.03	27.1 ± 0.8	26.9 ± 0.8	28.2 ± 0.8	27.8 ± 0.8
	12	0.82 ± 0.01	0.69 ± 0.01	0.64 ± 0.01	0.30 ± 0.01	2.14 ± 0.04	1.84 ± 0.03	1.79 ± 0.03	0.82 ± 0.01	27.7 ± 0.8	27.4 ± 0.8	26.4 ± 0.7	27.0 ± 0.6
	18	0.78 ± 0.01	0.64 ± 0.01	0.55 ± 0.01	0.22 ± 0.01	2.02 ± 0.04	1.73 ± 0.03	1.62 ± 0.03	0.55 ± 0.01	28.0 ± 0.8	27.1 ± 0.7	25.5 ± 0.7	28.8 ± 0.6
	24	0.74 ± 0.01	0.54 ± 0.01	0.45 ± 0.01	0.15 ± 0.01	1.83 ± 0.03	1.49 ± 0.03	1.20 ± 0.02	0.40 ± 0.01	28.9 ± 0.8	26.6 ± 0.7	27.3 ± 0.7	27.3 ± 0.6
Styrian Gold cones	0	1.86 ± 0.03	1.86 ± 0.03	1.86 ± 0.03	1.86 ± 0.03	3.60 ± 0.06	3.60 ± 0.06	3.60 ± 0.06	3.60 ± 0.06	34.0 ± 1.2	34.0 ± 1.20	34.0 ± 1.2	34.0 ± 1.2
	6	1.47 ± 0.03	1.36 ± 0.02	1.39 ± 0.02	1.22 ± 0.02	2.82 ± 0.05	2.63 ± 0.05	2.70 ± 0.05	2.36 ± 0.04	34.3 ± 1.1	34.1 ± 1.1	33.9 ± 1.1	34.0 ± 1.0
	12	1.38 ± 0.02	1.18 ± 0.02	1.27 ± 0.02	0.71 ± 0.01	2.67 ± 0.05	2.27 ± 0.04	2.42 ± 0.04	1.42 ± 0.03	34.1 ± 1.1	34.2 ± 1.0	34.3 ± 1.1	33.5 ± 0.9
	18	1.34 ± 0.02	1.13 ± 0.02	1.20 ± 0.02	0.56 ± 0.01	2.62 ± 0.05	2.22 ± 0.04	2.32 ± 0.04	1.10 ± 0.02	33.9 ± 1.1	33.8 ± 1.0	34.1 ± 1.0	33.8 ± 0.8
	24	1.20 ± 0.02	1.06 ± 0.02	0.99 ± 0.02	0.39 ± 0.01	2.31 ± 0.04	2.08 ± 0.04	1.96 ± 0.04	0.79 ± 0.01	34.2 ± 1.0	33.8 ± 1.0	33.6 ± 1.0	33.0 ± 0.7
Styrian Wolf cones	0	2.69 ± 0.05	2.69 ± 0.05	2.69 ± 0.05	2.69 ± 0.05	9.71 ± 0.17	9.71 ± 0.17	9.71 ± 0.17	9.71 ± 0.17	21.7 ± 1.2	21.7 ± 1.2	21.7 ± 1.2	21.7 ± 1.2
	6	2.47 ± 0.04	2.32 ± 0.04	1.92 ± 0.03	1.43 ± 0.02	8.96 ± 0.16	8.16 ± 0.15	6.77 ± 0.12	5.02 ± 0.09	21.6 ± 1.1	22.2 ± 1.1	22.1 ± 1.0	22.2 ± 0.9
	12	2.34 ± 0.04	2.13 ± 0.04	1.68 ± 0.03	0.88 ± 0.02	8.35 ± 0.15	7.71 ± 0.14	5.94 ± 0.11	2.96 ± 0.05	21.9 ± 1.1	21.7 ± 1.1	22.0 ± 1.0	22.9 ± 0.8
	18	2.23 ± 0.04	1.89 ± 0.03	1.38 ± 0.02	0.58 ± 0.01	7.72 ± 0.14	6.77 ± 0.12	4.90 ± 0.09	2.01 ± 0.04	22.4 ± 1.1	21.9 ± 1.0	22.0 ± 0.9	22.3 ± 0.6
	24	1.99 ± 0.03	1.79 ± 0.03	1.04 ± 0.02	0.45 ± 0.01	7.09 ± 0.13	6.32 ± 0.11	3.66 ± 0.07	1.68 ± 0.03	21.9 ± 1.0	22.1 ± 1.0	22.2 ± 0.8	21.2 ± 0.6

Table S2: The content of co-humulone. (n+ad)-humulone and % of co-humulone in alpha-acids in hop pellets.

	time	co-humulone				(n+ad)-humulone				% co-humulone in alpha-acids			
Variety/Conditions		A	B	C	D	A	B	C	D	A	B	C	D
Celeia pellets	0	0.79 ± 0.01	0.79 ± 0.01	0.79 ± 0.01	0.79 ± 0.01	2.39 ± 0.04	2.39 ± 0.04	2.39 ± 0.04	2.39 ± 0.04	24.8 ± 0.8	24.8 ± 0.8	24.9 ± 0.8	24.9 ± 0.8
	6	0.71 ± 0.01	0.69 ± 0.01	0.62 ± 0.01	0.62 ± 0.01	2.00 ± 0.04	1.95 ± 0.03	1.76 ± 0.03	1.76 ± 0.03	26.3 ± 0.8	26.1 ± 0.7	26.0 ± 0.7	26.1 ± 0.7
	12	0.70 ± 0.01	0.53 ± 0.01	0.55 ± 0.01	0.30 ± 0.01	1.95 ± 0.03	1.56 ± 0.03	1.54 ± 0.03	0.84 ± 0.02	26.3 ± 0.8	25.5 ± 0.7	26.2 ± 0.7	26.2 ± 0.6
	18	0.67 ± 0.01	0.51 ± 0.01	0.51 ± 0.01	0.24 ± 0.01	1.90 ± 0.03	1.49 ± 0.03	1.47 ± 0.03	0.73 ± 0.01	26.2 ± 0.7	25.4 ± 0.7	25.9 ± 0.7	25.0 ± 0.6
	24	0.63 ± 0.01	0.40 ± 0.01	0.35 ± 0.01	0.07 ± 0.01	1.76 ± 0.03	1.15 ± 0.02	1.02 ± 0.02	0.21 ± 0.01	26.5 ± 0.7	25.5 ± 0.6	25.5 ± 0.6	24.0 ± 0.5
Auora pellets	0	2.55 ± 0.04	2.55 ± 0.04	2.55 ± 0.04	2.55 ± 0.04	8.77 ± 0.16	8.77 ± 0.16	8.77 ± 0.16	8.77 ± 0.16	22.5 ± 1.2	22.5 ± 1.2	22.5 ± 1.2	22.5 ± 1.2
	6	2.42 ± 0.04	2.22 ± 0.04	2.21 ± 0.04	2.13 ± 0.04	8.41 ± 0.15	7.61 ± 0.14	7.53 ± 0.13	7.31 ± 0.13	22.4 ± 1.1	22.6 ± 1.1	22.7 ± 1.1	22.5 ± 1.1
	12	2.35 ± 0.04	2.08 ± 0.04	2.10 ± 0.04	1.67 ± 0.03	8.02 ± 0.14	7.11 ± 0.13	7.22 ± 0.13	5.66 ± 0.10	22.6 ± 1.1	22.6 ± 1.1	22.5 ± 1.1	22.8 ± 1.0
	18	2.29 ± 0.04	2.01 ± 0.03	1.94 ± 0.03	1.45 ± 0.03	7.78 ± 0.14	6.99 ± 0.13	6.54 ± 0.12	4.99 ± 0.09	22.7 ± 1.1	22.3 ± 1.0	22.9 ± 1.0	22.5 ± 0.9
	24	2.07 ± 0.04	1.86 ± 0.03	1.70 ± 0.03	0.91 ± 0.02	6.98 ± 0.12	6.49 ± 0.12	5.76 ± 0.10	3.22 ± 0.06	22.9 ± 1.1	22.3 ± 1.0	22.8 ± 1.0	22.0 ± 0.8
Bobek pellets	0	1.43 ± 0.02	1.43 ± 0.02	1.43 ± 0.02	1.43 ± 0.02	4.71 ± 0.08	4.71 ± 0.08	4.71 ± 0.08	4.71 ± 0.08	22.5 ± 0.9	23.3 ± 0.9	23.3 ± 0.9	23.3 ± 0.9
	6	1.26 ± 0.02	1.06 ± 0.02	1.08 ± 0.02	0.82 ± 0.01	4.04 ± 0.07	3.48 ± 0.06	3.44 ± 0.06	2.63 ± 0.05	23.7 ± 0.9	23.3 ± 0.8	23.9 ± 0.8	23.8 ± 0.8
	12	1.17 ± 0.02	0.95 ± 0.02	0.99 ± 0.02	0.11 ± 0.01	3.8 ± 0.07	3.10 ± 0.06	3.22 ± 0.06	0.45 ± 0.01	23.6 ± 0.9	23.4 ± 0.8	23.6 ± 0.8	19.6 ± 0.4
	18	1.15 ± 0.02	0.90 ± 0.02	0.93 ± 0.02	0.05 ± 0.01	3.69 ± 0.07	2.92 ± 0.05	3.05 ± 0.05	0.21 ± 0.01	23.7 ± 0.9	23.6 ± 0.8	23.4 ± 0.8	20.8 ± 0.4
	24	1.00 ± 0.02	0.79 ± 0.01	0.85 ± 0.01	0.02 ± 0.01	3.20 ± 0.06	2.62 ± 0.05	2.74 ± 0.05	0.05 ± 0.01	23.8 ± 0.8	23.1 ± 0.7	23.7 ± 0.8	28.6 ± 0.5
Styrian Golding pellets	0	1.08 ± 0.02	1.08 ± 0.02	1.08 ± 0.02	1.08 ± 0.02	2.58 ± 0.05	2.58 ± 0.05	2.58 ± 0.05	2.58 ± 0.05	29.5 ± 0.9	29.5 ± 0.9	29.5 ± 0.9	29.5 ± 0.9
	6	0.87 ± 0.02	0.77 ± 0.01	0.79 ± 0.01	0.76 ± 0.01	2.10 ± 0.04	1.83 ± 0.03	1.92 ± 0.03	1.83 ± 0.03	29.5 ± 0.9	29.6 ± 0.8	29.2 ± 0.8	29.3 ± 0.8
	12	0.83 ± 0.01	0.68 ± 0.01	0.77 ± 0.01	0.54 ± 0.01	2.01 ± 0.04	1.63 ± 0.03	1.83 ± 0.03	1.37 ± 0.02	29.1 ± 0.8	29.4 ± 0.8	29.5 ± 0.8	28.1 ± 0.7
	18	0.81 ± 0.01	0.65 ± 0.01	0.73 ± 0.01	0.45 ± 0.01	1.95 ± 0.03	1.58 ± 0.03	1.78 ± 0.03	1.18 ± 0.02	29.4 ± 0.8	29.2 ± 0.8	29.1 ± 0.8	27.7 ± 0.7
	24	0.79 ± 0.01	0.54 ± 0.01	0.67 ± 0.01	0.25 ± 0.01	1.80 ± 0.03	1.40 ± 0.03	1.59 ± 0.03	0.72 ± 0.01	30.4 ± 0.8	27.8 ± 0.7	30.7 ± 0.8	26.1 ± 0.6
Styrian Wolf pellets	0	3.01 ± 0.05	3.01 ± 0.05	3.01 ± 0.05	3.01 ± 0.05	10.1 ± 0.18	10.1 ± 0.18	10.1 ± 0.18	10.1 ± 0.18	23.0 ± 1.3	23.0 ± 1.3	23.0 ± 1.3	23.0 ± 1.3
	6	2.78 ± 0.05	2.61 ± 0.05	2.17 ± 0.04	1.70 ± 0.03	9.44 ± 0.17	8.83 ± 0.16	7.38 ± 0.13	5.74 ± 0.10	22.7 ± 1.2	22.8 ± 1.2	22.8 ± 1.1	22.9 ± 1.0
	12	2.74 ± 0.05	2.43 ± 0.04	1.91 ± 0.03	0.87 ± 0.02	9.31 ± 0.17	8.34 ± 0.15	6.44 ± 0.12	3.00 ± 0.05	22.7 ± 1.2	22.6 ± 1.1	22.9 ± 1.0	22.4 ± 0.7
	18	2.71 ± 0.05	2.31 ± 0.04	1.54 ± 0.03	0.60 ± 0.01	9.13 ± 0.16	7.89 ± 0.14	5.16 ± 0.09	2.10 ± 0.04	22.9 ± 1.2	22.6 ± 1.1	23.0 ± 0.9	22.2 ± 0.7
	24	2.62 ± 0.05	2.09 ± 0.04	1.05 ± 0.02	0.16 ± 0.01	8.94 ± 0.16	7.15 ± 0.13	3.55 ± 0.06	0.56 ± 0.01	22.7 ± 1.2	22.6 ± 1.1	22.8 ± 0.8	22.4 ± 0.5

Table S3: The content of co-lupulone. (n+ad)-lupulone and % of co-lupulone in beta-acids in hop cones.

	time	co-lupulone				(n+ad)-lupulone				% co-lupulone in beta-acids			
Variety/Conditions		A	B	C	D	A	B	C	D	A	B	C	D
Celeia cones	0	1.88 ± 0.03	1.88 ± 0.03	1.88 ± 0.03	1.88 ± 0.03	1.30 ± 0.02	1.30 ± 0.02	1.30 ± 0.02	1.30 ± 0.02	59.2 ± 1.4	59.2 ± 1.4	59.1 ± 1.4	59.2 ± 1.4
	6	1.55 ± 0.02	1.41 ± 0.02	1.33 ± 0.02	1.04 ± 0.02	1.05 ± 0.02	0.98 ± 0.02	0.92 ± 0.01	0.70 ± 0.01	59.6 ± 1.4	58.9 ± 1.3	59.1 ± 1.3	59.9 ± 1.2
	12	1.52 ± 0.02	1.25 ± 0.02	1.06 ± 0.02	0.42 ± 0.01	1.00 ± 0.02	0.84 ± 0.01	0.70 ± 0.01	0.29 ± 0.01	60.2 ± 1.4	59.8 ± 1.3	60.2 ± 1.2	59.4 ± 1.0
	18	1.44 ± 0.02	1.17 ± 0.02	0.93 ± 0.01	0.32 ± 0.01	0.95 ± 0.02	0.79 ± 0.01	0.62 ± 0.01	0.18 ± 0.01	60.3 ± 1.4	59.7 ± 1.3	60.1 ± 1.2	64.4 ± 1.1
	24	1.29 ± 0.02	0.92 ± 0.01	0.66 ± 0.01	0.18 ± 0.01	0.85 ± 0.01	0.61 ± 0.01	0.46 ± 0.01	0.11 ± 0.01	60.3 ± 1.3	60.1 ± 1.2	59.0 ± 1.1	61.5 ± 1.0
Auora cones	0	2.53 ± 0.04	2.53 ± 0.04	2.53 ± 0.04	2.53 ± 0.04	2.38 ± 0.04	2.38 ± 0.04	2.38 ± 0.04	2.38 ± 0.04	51.6 ± 1.5	51.6 ± 1.5	51.6 ± 1.5	51.6 ± 1.5
	6	2.34 ± 0.03	2.14 ± 0.03	2.10 ± 0.03	1.91 ± 0.03	2.20 ± 0.04	2.03 ± 0.03	1.95 ± 0.03	1.79 ± 0.03	51.6 ± 1.4	51.8 ± 1.4	51.6 ± 1.4	51.8 ± 1.3
	12	2.26 ± 0.03	2.04 ± 0.03	1.84 ± 0.03	0.98 ± 0.01	2.09 ± 0.03	1.95 ± 0.03	1.74 ± 0.03	0.89 ± 0.01	51.9 ± 1.4	51.4 ± 1.4	52.4 ± 1.3	51.8 ± 1.1
	18	2.21 ± 0.03	1.98 ± 0.03	1.72 ± 0.03	0.63 ± 0.01	2.03 ± 0.03	1.87 ± 0.03	1.59 ± 0.03	0.53 ± 0.01	52.1 ± 1.4	52.0 ± 1.4	54.3 ± 1.3	52.3 ± 1.0
	24	1.90 ± 0.03	1.67 ± 0.02	1.51 ± 0.02	0.31 ± 0.01	1.78 ± 0.03	1.62 ± 0.03	1.37 ± 0.02	0.23 ± 0.01	51.7 ± 1.3	52.5 ± 1.3	57.1 ± 1.4	52.0 ± 0.9
Bobek cones	0	2.45 ± 0.04	2.45 ± 0.04	2.45 ± 0.04	2.45 ± 0.04	2.33 ± 0.04	2.33 ± 0.04	2.33 ± 0.04	2.33 ± 0.04	51.3 ± 1.4	51.3 ± 1.4	51.3 ± 1.4	51.3 ± 1.4
	6	2.16 ± 0.03	1.93 ± 0.03	1.72 ± 0.03	1.79 ± 0.03	2.12 ± 0.03	1.89 ± 0.03	1.71 ± 0.03	1.77 ± 0.03	50.5 ± 1.4	50.5 ± 1.3	50.2 ± 1.3	50.3 ± 1.3
	12	2.00 ± 0.03	1.66 ± 0.02	1.57 ± 0.02	0.60 ± 0.01	1.96 ± 0.03	1.68 ± 0.03	1.52 ± 0.02	0.58 ± 0.01	50.6 ± 1.3	49.7 ± 1.2	50.9 ± 1.2	50.6 ± 1.0
	18	1.93 ± 0.03	1.58 ± 0.02	1.29 ± 0.02	0.45 ± 0.01	1.88 ± 0.03	1.58 ± 0.03	1.30 ± 0.02	0.38 ± 0.01	50.7 ± 1.3	50.0 ± 1.2	49.8 ± 1.1	53.7 ± 1.0
	24	1.75 ± 0.03	1.34 ± 0.02	1.04 ± 0.02	0.24 ± 0.01	1.75 ± 0.03	1.29 ± 0.02	1.01 ± 0.02	0.21 ± 0.01	50.0 ± 1.3	50.8 ± 1.2	50.5 ± 1.1	53.7 ± 0.9
Styrian Gold cones	0	2.96 ± 0.04	2.96 ± 0.04	2.96 ± 0.04	2.96 ± 0.04	1.84 ± 0.03	1.84 ± 0.03	1.84 ± 0.03	1.84 ± 0.03	61.7 ± 1.7	61.7 ± 1.7	61.7 ± 1.7	61.7 ± 1.7
	6	2.35 ± 0.03	2.09 ± 0.03	2.13 ± 0.03	1.81 ± 0.03	1.45 ± 0.02	1.37 ± 0.02	1.35 ± 0.02	1.15 ± 0.02	61.8 ± 1.6	60.3 ± 1.5	61.1 ± 1.5	61.0 ± 1.5
	12	2.22 ± 0.03	1.82 ± 0.03	1.98 ± 0.03	0.80 ± 0.01	1.39 ± 0.02	1.16 ± 0.02	1.25 ± 0.02	0.51 ± 0.01	61.5 ± 1.6	61.0 ± 1.5	61.3 ± 1.5	61.0 ± 1.2
	18	2.17 ± 0.03	1.75 ± 0.03	1.90 ± 0.03	0.64 ± 0.01	1.35 ± 0.02	1.13 ± 0.02	1.18 ± 0.02	0.39 ± 0.01	61.6 ± 1.6	60.9 ± 1.4	61.7 ± 1.5	62.4 ± 1.2
	24	2.05 ± 0.03	1.61 ± 0.02	1.53 ± 0.02	0.39 ± 0.01	1.22 ± 0.02	1.07 ± 0.02	0.97 ± 0.02	0.20 ± 0.01	62.6 ± 1.6	60.1 ± 1.4	61.2 ± 1.4	66.0 ± 1.1
Styrian Wolf cones	0	2.11 ± 0.03	2.11 ± 0.03	2.11 ± 0.03	2.11 ± 0.03	2.03 ± 0.03	2.03 ± 0.03	2.03 ± 0.03	2.03 ± 0.03	51.0 ± 1.4	51.0 ± 1.4	51.0 ± 1.4	51.0 ± 1.4
	6	1.81 ± 0.03	1.64 ± 0.02	1.42 ± 0.02	1.11 ± 0.02	1.75 ± 0.03	1.56 ± 0.02	1.38 ± 0.02	1.03 ± 0.02	50.9 ± 1.3	51.2 ± 1.3	50.9 ± 1.2	51.9 ± 1.1
	12	1.73 ± 0.03	1.44 ± 0.02	1.12 ± 0.02	0.39 ± 0.01	1.63 ± 0.03	1.29 ± 0.02	1.12 ± 0.02	0.35 ± 0.01	51.5 ± 1.3	52.7 ± 1.2	49.9 ± 1.1	52.7 ± 0.9
	18	1.63 ± 0.02	1.28 ± 0.02	0.90 ± 0.01	0.21 ± 0.01	1.57 ± 0.03	1.27 ± 0.02	0.94 ± 0.02	0.18 ± 0.01	50.9 ± 1.2	50.2 ± 1.1	49.0 ± 1.0	53.8 ± 0.9
	24	1.38 ± 0.02	1.17 ± 0.02	0.63 ± 0.01	0.15 ± 0.01	1.31 ± 0.02	1.09 ± 0.02	0.57 ± 0.01	0.11 ± 0.01	51.2 ± 1.2	51.6 ± 1.1	52.3 ± 1.0	57.7 ± 0.9

Table S4: The content of co-lupulone. (n+ad)-lupulone and % of co-lupulone in beta-acids in hop pellets.

	time	co-lupulone				(n+ad)-lupulone				% co-lupulone in beta-acids			
Variety/Conditions		A	B	C	D	A	B	C	D	A	B	C	D
Celeia pellets	0	1.81 ± 0.03	1.81 ± 0.03	1.81 ± 0.03	1.81 ± 0.03	1.34 ± 0.02	1.34 ± 0.02	1.34 ± 0.02	1.34 ± 0.02	57.4 ± 1.4	57.4 ± 1.4	57.4 ± 1.4	57.4 ± 1.4
	6	1.60 ± 0.02	1.55 ± 0.02	1.38 ± 0.02	1.40 ± 0.02	1.18 ± 0.02	1.13 ± 0.02	1.02 ± 0.02	1.04 ± 0.02	57.4 ± 1.3	57.9 ± 1.3	57.4 ± 1.3	57.4 ± 1.3
	12	1.54 ± 0.02	1.48 ± 0.02	1.28 ± 0.02	1.04 ± 0.02	1.11 ± 0.02	1.02 ± 0.02	0.93 ± 0.01	0.61 ± 0.01	58.0 ± 1.3	59.1 ± 1.3	58.1 ± 1.3	63.1 ± 1.3
	18	1.48 ± 0.02	1.42 ± 0.02	1.24 ± 0.02	1.00 ± 0.01	1.09 ± 0.02	0.99 ± 0.02	0.86 ± 0.01	0.53 ± 0.01	57.5 ± 1.3	58.8 ± 1.3	59.2 ± 1.3	65.2 ± 1.3
	24	1.30 ± 0.02	1.35 ± 0.02	1.00 ± 0.01	0.59 ± 0.01	0.97 ± 0.02	0.9 ± 0.01	0.60 ± 0.01	0.23 ± 0.01	57.4 ± 1.3	60.0 ± 1.3	62.5 ± 1.3	72.0 ± 1.3
Auora pellets	0	2.48 ± 0.04	2.48 ± 0.04	2.48 ± 0.04	2.48 ± 0.04	2.24 ± 0.04	2.24 ± 0.04	2.24 ± 0.04	2.24 ± 0.04	52.6 ± 1.5	52.6 ± 1.5	52.6 ± 1.5	52.6 ± 1.5
	6	2.35 ± 0.03	2.26 ± 0.03	2.14 ± 0.03	2.06 ± 0.03	2.15 ± 0.03	2.1 ± 0.03	1.97 ± 0.03	1.87 ± 0.03	52.2 ± 1.4	51.8 ± 1.4	52.1 ± 1.4	52.4 ± 1.4
	12	2.27 ± 0.03	2.18 ± 0.03	2.03 ± 0.03	1.93 ± 0.03	2.02 ± 0.03	2.02 ± 0.03	1.84 ± 0.03	1.76 ± 0.03	52.9 ± 1.4	51.8 ± 1.4	52.5 ± 1.4	52.2 ± 1.3
	18	2.15 ± 0.03	2.16 ± 0.03	1.98 ± 0.03	1.85 ± 0.03	1.94 ± 0.03	1.97 ± 0.03	1.79 ± 0.03	1.62 ± 0.03	52.5 ± 1.4	52.3 ± 1.4	52.5 ± 1.4	53.3 ± 1.3
	24	1.90 ± 0.03	1.96 ± 0.03	1.81 ± 0.03	1.43 ± 0.02	1.79 ± 0.03	1.81 ± 0.03	1.57 ± 0.03	1.13 ± 0.02	51.5 ± 1.3	52.0 ± 1.3	53.5 ± 1.3	55.9 ± 1.3
Bobek pellets	0	2.39 ± 0.04	2.39 ± 0.04	2.39 ± 0.04	2.39 ± 0.04	2.56 ± 0.04	2.56 ± 0.04	2.56 ± 0.04	2.56 ± 0.04	52.6 ± 1.5	48.2 ± 1.4	48.2 ± 1.4	48.2 ± 1.4
	6	2.16 ± 0.03	2.05 ± 0.03	1.97 ± 0.03	1.62 ± 0.02	2.39 ± 0.04	2.30 ± 0.04	2.18 ± 0.03	1.77 ± 0.03	47.5 ± 1.3	47.1 ± 1.3	47.4 ± 1.3	47.8 ± 1.2
	12	2.11 ± 0.03	1.96 ± 0.03	1.86 ± 0.03	0.39 ± 0.01	2.27 ± 0.04	2.15 ± 0.03	2.03 ± 0.03	0.31 ± 0.01	48.2 ± 1.3	47.8 ± 1.3	47.8 ± 1.3	55.6 ± 1.0
	18	2.02 ± 0.03	1.87 ± 0.03	1.83 ± 0.03	0.19 ± 0.01	2.16 ± 0.03	2.07 ± 0.03	2.01 ± 0.03	0.08 ± 0.01	48.4 ± 1.3	47.4 ± 1.2	47.6 ± 1.2	70.8 ± 1.1
	24	1.86 ± 0.03	1.75 ± 0.03	1.73 ± 0.03	0.05 ± 0.01	1.90 ± 0.03	1.93 ± 0.03	1.92 ± 0.03	0.02 ± 0.01	49.4 ± 1.3	47.6 ± 1.2	47.3 ± 1.2	71.4 ± 1.1
Styrian Gold pellets	0	1.40 ± 0.02	1.40 ± 0.02	1.40 ± 0.02	1.40 ± 0.02	1.14 ± 0.02	1.14 ± 0.02	1.14 ± 0.02	1.14 ± 0.02	55.0 ± 1.3	55 ± 1.3	55.0 ± 1.3	55.0 ± 1.3
	6	1.11 ± 0.02	1.00 ± 0.01	1.02 ± 0.02	0.97 ± 0.01	0.98 ± 0.02	0.87 ± 0.01	0.93 ± 0.01	0.87 ± 0.01	53.1 ± 1.1	53.4 ± 1.1	52.5 ± 1.1	52.6 ± 1.1
	12	1.05 ± 0.02	0.97 ± 0.01	0.97 ± 0.01	0.86 ± 0.01	0.92 ± 0.01	0.85 ± 0.01	0.86 ± 0.01	0.75 ± 0.01	53.1 ± 1.1	53.3 ± 1.1	53.0 ± 1.1	53.2 ± 1.1
	18	1.02 ± 0.02	0.96 ± 0.01	0.95 ± 0.01	0.83 ± 0.01	0.91 ± 0.01	0.83 ± 0.01	0.84 ± 0.01	0.70 ± 0.01	53.0 ± 1.1	53.7 ± 1.1	53.1 ± 1.1	54.3 ± 1.1
	24	0.92 ± 0.01	0.91 ± 0.01	0.89 ± 0.01	0.58 ± 0.01	0.8 ± 0.01	0.8 ± 0.01	0.81 ± 0.01	0.44 ± 0.01	53.5 ± 1.1	53.2 ± 1.1	52.3 ± 1.1	57.0 ± 1.1
Styrian Wolf pellets	0	2.19 ± 0.03	2.19 ± 0.03	2.19 ± 0.03	2.19 ± 0.03	2.05 ± 0.03	2.05 ± 0.03	2.05 ± 0.03	2.05 ± 0.03	51.7 ± 1.4	51.7 ± 1.4	51.7 ± 1.4	51.7 ± 1.4
	6	2.11 ± 0.03	2.02 ± 0.03	1.61 ± 0.02	1.28 ± 0.02	1.95 ± 0.03	1.91 ± 0.03	1.49 ± 0.02	1.15 ± 0.02	51.9 ± 1.4	51.4 ± 1.3	51.9 ± 1.3	52.6 ± 1.2
	12	2.05 ± 0.03	1.95 ± 0.03	1.35 ± 0.02	0.66 ± 0.01	1.91 ± 0.03	1.8 ± 0.03	1.24 ± 0.02	0.53 ± 0.01	51.8 ± 1.4	52.1 ± 1.3	52.1 ± 1.2	55.6 ± 1.1
	18	2.02 ± 0.03	1.88 ± 0.03	1.05 ± 0.02	0.44 ± 0.01	1.88 ± 0.03	1.75 ± 0.03	0.95 ± 0.02	0.34 ± 0.01	51.8 ± 1.4	51.7 ± 1.3	52.5 ± 1.1	56.3 ± 1.0
	24	1.95 ± 0.03	1.82 ± 0.03	0.69 ± 0.01	0.18 ± 0.01	1.84 ± 0.03	1.69 ± 0.03	0.59 ± 0.01	0.10 ± 0.01	51.5 ± 1.3	51.8 ± 1.3	53.8 ± 1.0	65.4 ± 1.0
